# Closing in on life: proximity dependent methods for life sciences

**DOI:** 10.18632/oncotarget.4890

**Published:** 2015-07-17

**Authors:** Björn Koos, Ola Söderberg

**Affiliations:** Department of Immunology, Genetics and Pathology, Science for Life Laboratory, Uppsala University, Biomedical Center, Uppsala, Sweden

**Keywords:** Chromosome Section, Hybridization chain reaction (HCR), Proximity ligation assay (PLA), Protein-protein interactions, Signaling

Method development is a corner stone in science, providing the tools required to address gaps in knowledge. For life sciences, methods for genomics and proteomics have facilitated the endeavors to map the human genome and proteome. These achievements have and will continue to profoundly influence our understanding of life and diseases, providing the blue print on cells and how the genetic information is translated into biological processes in a cell. For a multicellular organism, as ourselves, information at a single cell level is fundamental for the understanding of how individual cells are organized into organs, and how this is disrupted in malignancies. Hence, tools that preserve information on tissue architecture while obtaining information on activity status of each individual cell will be needed to determine intercellular communication and response to the microenvironment [1]. To analyze functional states of cellular signaling networks protein-protein interactions and post-translational modifications of proteins will have to be assayed, and to achieve this on endogenous proteins, affinity reagents, e.g. antibodies, will have to be used. The next task is to determine if the antibodies are in close proximity, i.e. pairwise binding to epitopes on interacting proteins, or a post-translational modification in combination with an epitope on the protein. By labeling antibodies or proteins with different fluorophores, Förster resonance energy transfer can be applied, monitoring changes in emission spectra from donor and acceptor fluorophores, or changes in lifetime [2].

Another approach to generate a signal, as a consequence of dual binding of antibodies, is to use DNA as reporter molecules. An advantage with DNA is the abundance of enzymes that can be used to modify and copy it, such as restriction enzymes, ligases and polymerases, and that hybridization between different oligonucleotides can be well predicted, which facilitates the design of the oligonucleotides used in an assay [3]. The *in situ* proximity ligation assay (*in situ* PLA) [4] utilizes DNA-conjugated antibodies (proximity probes) as hybridization templates for a pair of circularization oligonucleotides. Addition of a DNA-ligase will create a circular DNA molecule, as a surrogate marker for the pairwise binding of the proximity probes. This circle can then be amplified by phi29 DNA-polymerase, using the oligonucleotide on the proximity probe as a primer. The rolling circle amplification (RCA) product will be included as an extension of the proximity probe and can be visualized by hybridization of fluorophore-labeled oligonucleotides, complementary to the RCA product. As each RCA product consists of several hundreds repeats, single RCA products are easily visible by regular epifluorescence or bright-field microscopy.

The hybridization chain reaction (HCR), developed by Dirks and Pierce [5], is another way of generating an amplification product without any enzymatic steps. The method is based on partially complementary oligonucleotides with hairpin structures that are kinetically trapped as monomers. In the easiest setting two hairpin species (H1 and H2) are used. Once an initiator oligonucleotide is introduced, it will invade one molecule of the first species of hairpins (H1). This will open up the hairpin so that it can invade one of the complementary hairpins (H2), which then can invade one of the first species again, and so on. The chain reaction is driven by release of potential energy stored in the hairpin structure. The amplification method has since been used for many applications and can be used for parallel analysis of several targets [6].

We were inspired by the beauty of HCR and set out to develop a method combining HCR with the proximity dependence of *in situ* PLA, to obtain a method for analysis of protein-protein interactions and post-translational modifications that would not need any enzymatic steps. The method was described in the 12th of June issue of *Nature Communications*, and was named proxHCR [7]. The proximity probes in this method consist of DNA hairpins conjugated to antibodies. Once they have bound their targets an activator oligonucleotide will be added that invades one of the proximity probes. The opened proximity probe can then invade the second proximity probe, only if this is in close proximity. The bridged pair of proximity probes will exhibit the initiator sequence that will start HCR. As no enzymes are needed proxHCR is an inexpensive and robust alternative to *in situ* PLA, advantages that will make the method more appealing for less well funded laboratories throughout the world and could be exploited in automated staining procedures and point-of-care devices.
